# Characterization of Rat ILCs Reveals ILC2 as the Dominant Intestinal Subset

**DOI:** 10.3389/fimmu.2020.00255

**Published:** 2020-02-19

**Authors:** Ahmed Abidi, Thomas Laurent, Gaëlle Bériou, Laurence Bouchet-Delbos, Cynthia Fourgeux, Cédric Louvet, Raja Triki-Marrakchi, Jeremie Poschmann, Régis Josien, Jérôme Martin

**Affiliations:** ^1^Université de Nantes, Inserm, CHU Nantes, Centre de Recherche en Transplantation et Immunologie, UMR 1064, ITUN, Nantes, France; ^2^Université de Tunis El Manar, Laboratoire de Génétique, Immunologie et Pathologies Humaines, Faculté des Sciences de Tunis, Tunis, Tunisia; ^3^CHU Nantes, Laboratoire d'Immunologie, Nantes, France

**Keywords:** innate lymphoid cells, rat, secondary lymphoid organs, intestine, ILC2

## Abstract

Innate lymphoid cells (ILCs) are tissue-resident lymphocytes that lack antigen-specific receptors and exhibit innate effector functions such as cytokine production that play an important role in immediate responses to pathogens especially at mucosal sites. Mouse and human ILC subsets have been extensively characterized in various tissues and in blood. In this study, we present the first characterization of ILCs and ILC subsets in rat gut and secondary lymphoid organs using flow cytometry and single cell RNA sequencing. Our results show that phenotype and function of rat ILC subsets are conserved as compared to human and mouse ILCs. However, and in contrast to human and mouse, our study unexpectedly revealed that ILC2 and not ILC3 was the dominant ILC subset in the rat intestinal lamina propria. ILC2 predominance in the gut was independent of rat strain, sex or housing facility. In contrast, ILC3 was the predominant ILC subset in mesenteric lymph nodes and Peyer patches. In conclusion, our study demonstrates that in spite of highly conserved phenotype and function between mice, rat and humans, the distribution of ILC subsets in the intestinal mucosa is dependent on the species likely in response to both genetic and environmental factors.

## Introduction

Innate lymphoid cells (ILCs) are recombination-activating genes (RAGs)-independent lymphocytes that can be divided into three subsets, namely ILC1, ILC2, and ILC3, based on the cytokines they produce and the transcription factors (TFs) guiding their differentiation (1). ILCs are characterized by their capacity to respond rapidly to tissue damage and/or infection by producing sets of effector cytokines matching those of T helper cell subsets (2). T-bet^+^ ILC1 produce IFNγ in response to IL-12, IL-15, and IL-18 to promote the control of intracellular pathogens by macrophages (3). GATA-3^+^ ILC2 respond to IL-25, IL-33, and TSLP by producing key mediators of immune responses against helminths such as IL-5, IL-9, and IL-13 (4). Finally, RORγt^+^ ILC3 respond to myeloid cell-derived IL-23 and IL-1β and produce IL-17 and/or IL-22, which support epithelial defense mechanisms (5–7). ILC3 in tissues are further separated into at least two subsets: CCR6^+^ lymphoid tissue-inducer-like (LTi-like) ILC3 and natural cytotoxicity receptors positive ILC3 (NCR^+^ ILC3) (8). A subset of regulatory ILCs (ILCreg), which produces IL-10 and TGF-β, has been described recently. These cells do not express FOXP3 and have been implicated in the resolution of inflammation during colitis (9).

ILCs are mostly found in lymphoid organs and barrier tissues such as skin, lungs, and gut, where the relative subset distribution is influenced by local micro-environmental cues (10, 11). Accordingly, human and mouse studies showed that while ILC2 are more abundant before microbial colonization in the fetal intestine (6), ILC3 dominate over ILC2 and ILC1 during adulthood. Indeed, mouse ILC3 respond rapidly to the commensal microbiota upon colonization (12) and early studies revealed that subsets such as NKp46^+^ ILC3 proliferate after birth in the small intestine (13). Diet-derived metabolites such as retinoic acid also contribute to shape the intestinal ILC composition by supporting ILC3 development (14).

While ILCs have been the subject of extensive characterization in human and mouse tissues, almost no study has addressed ILC distribution and function in the rat. Recent evidence suggest that significant differences exist between rat and mouse microbiota composition that are associated with distinct cardinal features of their respective intestinal immune system, but whether it could include ILCs was not assessed (15, 16). The rat has been used as an animal model for physiology, pharmacology, toxicology, nutrition, behavior, immunology, and neoplasia for over 150 years (17, 18). Because rats are frequently used by immunologists in pre-clinical models of metabolic diseases and inflammatory bowel diseases (IBD) (19–21), a better understanding of rat intestinal ILCs is thus of particular importance.

In this study, we used flow cytometry and scRNAseq to characterize ILC subsets and cytokine production in rat intestinal tissues and secondary lymphoid organs. We confirmed ILC3 as the main ILC population in secondary lymphoid organs. However, ILC2 unexpectedly dominated the ILC composition of both ileum and colon lamina propria. ILC2 enrichment in intestinal effector sites was not dependent on the rat strain thus revealing major species differences between rat and mouse intestinal ILC distribution.

## Materials and Methods

### Rats

Ten to 14 weeks old female and male Sprague-Dawley (SPD), Lewis 1W (LEW) or Brown-Norway (BN) rats were used in this study. Animals were purchased from Centre d'Elevage Janvier (Le Genest-St Isle, France) or from our facility, and were kept under specific pathogen-free conditions. This study was conducted in accordance with the EU Directive 2010/63/EU for animal experiments, and the guidelines of the French Agriculture Ministry. This study was approved by the Veterinary Departmental Services committee (# E.44011).

### Reagents

Cells were cultured in complete Roswell Park Memorial Institute (RPMI) 1640 Medium (Invitrogen, Carlsbad, CA) supplemented with Golgi Stop (BD sciences, Le Pont de Claix, France). For ILC1 stimulation, 50 ng/ml of rat rIL-12 (Cat# 1760-RL) from R&D systems Europe (Lille, France) was used. For the ILC2 stimulation cocktail, human rTSLP, human rIL-33 and rat rIL-25 were purchased from Peprotech (Neuilly-sur-seine, France) and used at 50 ng/ml concentration. For ILC3 stimulation, 50 ng/ml of rat rIL-23 (Cat# 3136-RL) from R&D systems Europe (Lille, France), rat rIL-1β and human rTGFβ1 from Peprotech, human rIL-7 and 50 U/ml of human rIL-2 from Miltenyi Biotech (Paris, France), were used. PMA (Phorbol 12-myristate 13-acetate), Ionomycin, DNaseI, EDTA, SVF, Hank's balanced salt solution (HBSS), Collagenase D and Collagenase II were purchased from Sigma-Aldrich (Saint-Louis, MI); all reagents indicated are at the final concentration of use.

### Cell Isolation

#### Mesenteric Lymph Nodes Digestion Protocol

Mesenteric lymph nodes (MLN) were harvested, then dilacerated using 26G needles and digested in digestion solution containing RPMI 1640 Medium with 2 mg/ml of Collagenase D supplemented with 100 μg/ml of DNAseI for 30 min of shaking at 100 rpm and +37°C. Digestion was stopped 5 min before the end of cycle through the addition of Ethylenediaminetetraacetic acid (EDTA) (0.01 M). Cells were filtered on 100 μm cell strainer. Samples were centrifuged (10 min, 1,500 rpm, 4°C) and supernatant removed. MLN cells were resuspended in appropriate amount of FACS buffer containing 1X PBS supplemented with 2% FBS and 2% (0.1M) EDTA.

#### Peyer's Patches Digestion Protocol

Peyer's patches (PP) were first, washed in cold-PBS in a petri dish then transferred into 5 ml of solution 1 containing 1.5 mM DTT, 30 mM EDTA, and 1X PBS for 10 min incubation at 100 rpm +37°C. The suspension was then vortexed vigorously for 20 s and filtered through a 100 μm cell strainer. Then PPs were placed into 5 ml solution 2 (same as solution 1 but without DTT) and incubated in the same conditions. After washing, PPs were digested for 1 h in digestion solution at 100 rpm and +37°C. Finally, cells were filtered on 100 and 40 μm cells-strainers and suspended in FACS buffer.

#### Ileum and Colon Digestion Protocol

Ileum (without PP) and Colon were flushed with cold-PBS in petri dish after fat was removed. The tissues were opened longitudinally and cut into 0.5 cm pieces, then incubated once in 10 ml of solution 1 and twice with 10 ml solution 2 as described for PP to remove epithelial cells. Supernatant were discarded and the remaining tissues were digested twice in digestion solution for 30 min at 100 rpm and +37°C in 50 ml Falcon tubes. Finally, cells from the two digestions were pooled and filtered through a 100 and 40 μm cells-strainer and re-suspended in FACS buffer.

#### Spleen Digestion Protocol

Spleen were digested in digestion solution for 30 min with slow shaking (50 rpm) at 37°C in petri dish. Then, 0.01 M of EDTA was added for the last 5 min to stop the digestion. Next, red blood cells were lysed at room temperature and cells were then filtered through a 100 μm cell strainer. Samples were centrifuged for 10 min, 1,500 rpm at 4°C and resuspended in FACS buffer.

#### Lung Digestion Protocol

Lungs were cut into small fragments and digested in digestion solution for 30 min at 100 rpm and +37°C in 50 ml Falcon tubes. The remaining tissue fragments were smashed on 70 μm cell-strainer and washed with FACS buffer and centrifuged (10 min, 1,500 rpm, 4°C) and supernatant removed. Red blood cells were lysed at room temperature and cells were then filtered through a 70 μm cell strainer.

#### Adipose Tissue Digestion Protocol

Perigonadal and fat pads adipose tissues were used as representative visceral adipose tissue (VAT). Adipose tissues were finely dissected with a scalpel blade and digested in digestion solution containing RPMI 1640 Medium with 2 mg/ml of Collagenase II supplemented with 100 μg/ml of DNAseI for 30 min of shaking at 100 rpm and +37°C. Digestion tubes rested vertically 5 min at RT before discarding the upper phase containing remaining fat and adipocytes. The remaining digests were then filtered through 70 μm cell-strainers and centrifuged at 800 g for 15 min.

### Flow Cytometry

Surface staining was realized with antibodies diluted in FACS buffer at 4°C for 30 min. First, cells were stained with a lineage antibody cocktail-FITC [TCRαβ (R7/3, in house), TCRγδ (V65, in house), CD11b/c (OX42, in house), CD172α (OX41, in house), Igκ (OX12, in house), and CD3ε (G4.18, BD Biosciences)]; CD4-PE.Cy7 (OX35, BD Biosciences), CD45-APC.Cy7 (OX1, BD Biosciences), MHC-II-PerCp (OX6, BD Biosciences), CD127-PE (R&D systems), and NKRP1-Pacific blue (3.2.3, in house). Staining of NKp46-Pacific blue (Wen23, in house) was performed at room temperature for 45 min. Cells were fixed and stained intracellularly using the Foxp3 Staining Buffer kit (eBioscience, catalog # 00-5523-00) according to the manufacturer's instructions. For TFs staining, T-bet-APC (REA102), GATA-3-APC/Vio.Blue (REA174), and RORγt-APC (REA278) were purchased from Miltenyi Biotech (Paris, France). For cytokines detection, IFNγ-BB700 (DB1, BD Biosciences) IL-22-PerCp.Cy5.5 (Poly5164, BioLegend), IL-17A-APC (eBio17B7, eBioscience), and IL-13-APC (in house) mAb were used. For cell proliferation, Ki67-PE.Cy7 (B56, BD Biosciences) was used. Viability-Dye 506 was used to separate dead/live cells. Samples were run on the BD FACS Canto II™ (BD Biosciences) and data collected using BD FACSDiva Software (BD biosciences). For cells sorting, MLN were negatively enriched in ILCs with the use of depleting Abs (TCRαβ, Igκ, CD8, and CD11b/c, all in house).

### Real-Time Quantitative Reverse Transcriptase-PCR

Total RNA was isolated from total tissue and sorted Lin^−^ CD127^+^ cells from MLN of SPD rats using Trizol reagent (Invitrogen) according to the manufacturer's instructions. Reverse transcription was performed using Murine Moloney Leukemia Virus Reverse Transcriptase (Invitrogen) following to the manufacturer's instructions. *Rag1* gene expression level was determined by relative quantitative RT-PCR using Taqman probe (Thermo Fisher scientific) *Rag1*: Rn01484440_m1, reported to *Hprt1*: Rn01527840_m1 expression level. Taqman Fast advanced Master Mix 2X reagent mix was used (Applied Biosystem). RT-PCR was performed on the StepOne plus system (Applied Biosystem).

### Single Cell RNAseq Preparation and Analysis

scRNA-seq libraries were produced with the Chromium Single Cell 3′ Library and Gel Bead Kit v3 (10X Genomics, San Francisco, CA). Libraries were then sequenced on a rapid run flowcell (75 bp) with an Illumina NextSeq 500.

FASTQ files were demultiplexed with CellRanger v3.0.1 (10X Genomics) and aligned on the Rnor_6.0 rat reference genome. Six thousand and four hundred and fifty six cells were recovered with CellRanger and their gene expression matrices were loaded on R 3.6.2. Single cells had an average of 69,331 mapped reads per cell with a median of 4,565 unique molecular identifiers (UMI) and a median of 1,513 detected genes per cell.

Genes expressed in <3 cells were excluded as well as cells that had <300 expressed genes or <500 total UMIs. To remove potential cell doublets we excluded cells with more than 2,000 genes and 6,500 UMIs. Additionally, only cells with <5% of mitochondrial genes were kept for downstream analysis. The resulting count matrix was processed with the Seurat R package (22). Gene expression of single cells was then log-normalized with a scale factor of 10,000 to normalize the gene expression by the total expression of the cell. The top 2,000 most variable genes were selected afterwards to perform a linear dimensional reduction by principal components analysis (PCA). The 10 first principal components (PC) were used to identify cell clusters.

Two clusters of cells were excluded from the downstream analysis, as these clusters expressed well-known B cell markers (*Cd79a* and *Ms4a1*) and were likely contaminating B cells (4% of total cells) retained throughout the sorting. Finally, five clusters of cells were kept and visualized with a UMAP (23), a non-linear dimensionality reduction technique. ILC populations were annotated based on known markers and differentially expressed genes between clusters. Differential expression analysis to identify cluster-specific genes was performed with the Wilcoxon rank sum test and the DESeq2 package. Expression profiles were analyzed using scDissector an exploratory application (courtesy of Dr. Ephraim Kenigsberg, Icahn School of Medicine at Mount Sinai, New York, NY) (24).

### Statistical Analysis

Data collected were analyzed using Flow Jo software vX.6.7 (Treestar) and GraphPad Prism 6 (GraphPad Software, San Diego, CA). Significance was determined using the following non-parametric tests: Mann-Whitney test for comparison of two groups, and Tukey's test for comparison of more than two groups with 2 way ANOVA.

## Results

### Distribution of Innate Lymphoid Cells in Rat Spleen and Intestinal Tissues

**Figure 1:**
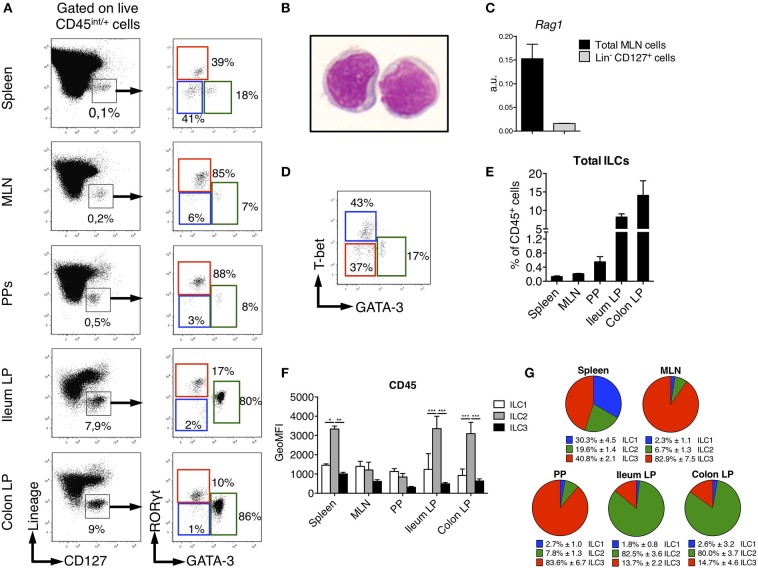
Identification of ILC subsets in SPD Rats. To characterize ILC subsets in lymphoid and gut-associated tissues, cells were isolated from mesenteric lymph nodes (MLN), Peyer's patches (PP), ileum and colon LP of SPD rats. **(A)** Representative flow cytometry plots showing the gating approach used to identify putative ILCs (Left panels) and ILC subsets (Right panels) on the basis of the expression of the transcription factors GATA-3 and RORγt in the depicted tissues. **(B)** May-Grünwald-Giemsa staining (original magnification X100) of rat Lin^−^ CD127^+^ ILCs as gated in **(A)** that were sort-purified from MLN of SPD rats. **(C)** *Rag1* gene expression was assessed by qPCR on total MLN cells and sorted Lin^−^ CD127^+^ cells SPD rats (*n* = 3). **(D)** Representative flow cytometric plot showing T-bet vs. GATA-3 expression in spleen ILC subsets from SPD rats. **(E)** Frequencies of total Lin^−^ CD127^+^ ILCs expressed as percentage of CD45^+^ leukocytes in different tissues from SPD rats. (*n* = 6). **(F)** CD45 mean fluorescence intensity (MFI) on ILC subsets from the depicted tissues from SPD rat. **(G)** Distribution of ILC subsets among total ILCs from SPD rats. *n* = 6 for each tissue. ^*^p < 0.05, ^**^p < 0.01, ^***^p < 0.001.

We sought to identify ILCs in rat SLOs (secondary lymphoid organs) and intestinal tissues. To limit biased cell-type frequencies, we did not perform any enrichment gradient before analysis. We defined rat ILCs by staining cells with a cocktail of mAb specific for B, T, and myeloid cells and CD127, which is highly expressed by human and mouse ILCs (25, 26) (Figure 1A). We included a staining of intracellular CD3ε in the lineage cocktail to avoid any potential T cell contamination of the ILC gate (27, 28). As in mice (29, 30), NK cells in SLOs did not express CD127 (Supplementary Figure 1). After gating on CD45^int/+^ cells to exclude stromal and residual epithelial cells, Lin^−^ CD127^+^ cells were clearly identified in all tissues analyzed (Figure 1A). May-Grünwald Giemsa staining of sorted Lin^−^ CD127^+^ cells confirmed their lymphoid morphology (Figure 1B). Almost no expression of recombination activating gene 1 (*Rag1*) was detected in Lin^−^ CD127^+^ cells as compared to total mesenteric lymph nodes (MLN) cells (Figure 1C). We assessed whether these candidate ILCs could be further separated based on their differential intracellular expression of the TFs GATA-3 and RORγt, which usually define human and mouse ILC2 and ILC3, respectively (31). In all tissues analyzed, we identified three populations of Lin^−^ CD127^+^ as GATA-3^−^ RORγt^−^, GATA-3^+^ RORγt^−/low^, and GATA-3^−^ RORγt^+^ cells, thus possibly corresponding to populations of ILC1, ILC2, and ILC3 (Figure 1A). Additionally, staining of T-bet and GATA-3 in the spleen also defined three populations of T-bet^+^ GATA-3^−^ (ILC1), T-bet^−^ GATA-3^+^ (ILC2), and T-bet^−^ GATA-3^−^ (ILC3) in proportions similar to those obtained with the GATA-3/RORγt staining (Figure 1D). Of note, ILC2 expressed intermediate levels of RORγt in the gut lamina propria (LP), Peyer's patches (PP) and MLN, but not in the spleen (Figure 1A). Because ILC1 were very rare and more loosely defined in intestinal tissues, we used the GATA-3/RORγt gating strategy to define ILC subsets in the rest of the study.

Rat ILCs were present at much higher frequency in the lamina propria of both ileum (9% of leukocytes) and colon (15%) as compared to spleen, PP and MLN (<1%) (Figure 1E). Consistent with human and mouse studies (32–35), rat ILC2 expressed higher levels of CD45 than ILC1 and ILC3 (Figure 1F). The relative enrichment of rat ILC subsets differed between the tissues analyzed, ILC3 being greatly enriched over ILC1 and ILC2 in MLN and PP, where they represented more than 80% of total ILCs. ILC3 were also the most abundant in spleen (40%) followed by ILC1 and ILC2 (Figure 1G). In sharp contrast, we observed an opposite distribution in ileum and colon LP where ILC2 largely dominated ILC composition (>80%), while ILC3 accounted for 15% of total ILCs and ILC1 were poorly represented.

### Unbiased Characterization of Colonic ILCs by Single-Cell RNA Sequencing

**Figure 2:**
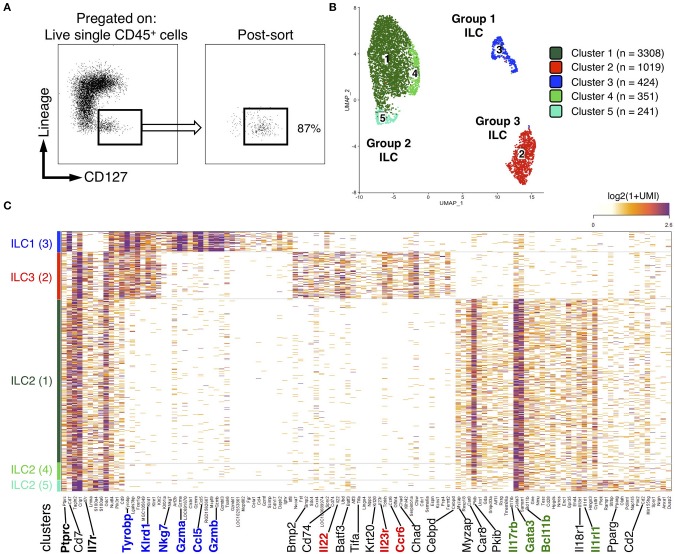
scRNAseq reveals distinct clusters of ILCs from colon LP of rat. **(A)** FACS-sorting plots showing the sorted CD45^+^ Lin^−^ CD127^+^ ILCs from the colon LP of one SPD rat (left panel) and cells purity after sorting (right panel). **(B)** Uniform manifold approximation and projection (UMAP) visualizing clusters of colonic Lin^−^ CD127^+^ cells analyzed by scRNAseq (*n* = 1 SPD rat). **(C)** Heatmap visualization color-coding the mRNA (UMI) counts per single cells (stacked rows) for selected genes (columns). Visualized are randomly selected cells, which were downsampled to 2,000 UMIs/cell. Clusters are separated by gray bars and ordered by ILC subtype.

ILC2 predominance in rat ileum and colon was rather unexpected, as it contrasted with ILC proportions described in human and mouse gut (36, 37). Because of the paucity of validated surface markers available to describe further rat ILCs by flow cytometry, we decided to unbiasedly characterize FACS-sorted Lin^−^ CD127^+^ cells using high-resolution scRNAseq (Figure 2A). We focused on colonic cells because of the unexpected high prevalence of ILC2 and the higher frequency of ILC in the colon as compared to the ileum (Figure 1A). We analyzed 5,343 single cells that passed QC (see methods). Clustering analysis revealed 5 distinct clusters (C1–C5), which were visualized using uniform manifold approximation and projection (UMAP) (Figure 2B). Three well-demarcated groups of clusters were identified and, respectively, comprised clusters C1, C4, and C5 (72% of total cells), C2 (20% of total cells), and C3 (8% of total cells) (Figure 2B). Cells in clusters C1, C4, and C5 expressed high levels of important ILC2 genes including the TFs *Gata3* and *Bcl11b*, as well as the receptors *Il1rl1* (ST2) and *Il17rb*, and were thus annotated as ILC2 (38, 39) (Figure 2C). The C2 cluster contained cells expressing strong levels of genes well-characterized in ILC3 such as *Nfil3, Tifa, Il23r*, and the cytokine *Il22*. The high expression of the chemokine receptor *CCR6* suggested colonic ILC3 were mostly LTi-like cells (40, 41) (Figure 2C and Supplementary Figure 2B). Finally, cells in cluster C3 expressed high levels of genes associated with NK cells such as *Tyrobp* (DAP12), *Irf8, Nkg7, Fcer1g, Il2rb* (CD122), *Ccl5, Gzma, Gzmb, Gzmm, Prf1*. Almost no cell in cluster C3 expressed the pan-ILC marker *Il7r* (CD127) (Figure 2C). No NK-specific mAb could be included in the lineage cocktail, and examination of CD127 expression confirmed the presence of contaminating CD127^−^ cells in sorted cells (Figure 2A, right panel). This suggested that NK cells in fact composed a large part of cluster C3. We thus chose to refer to these cells as group 1 ILCs to encompass both NK cells and rare ILC1 (Figure 2B and Supplementary Figures 2A,B). Deeper analysis of ILC2 revealed additional transcriptomic heterogeneity between cells in the 3 clusters. Cells in cluster C4 mostly differed by their high expression of heat-shock protein encoding genes such as *Dnajb1, Dnaja1, Hsph1, Hspd1*, and *Hspe1*, while cells in cluster C5 expressed higher levels of *Ramp1, Plek, S100a4, Tgfb1, Il18r1, Anxa1*, and the cytokine *Il5*, suggesting a possible higher activation state (Supplementary Figure 2C). Taken together, these data confirmed that ILC2 dominated the rat ILC landscape in the colon and suggested that the rare cells identified as ILC1 by our flow cytometry analysis could account for a mixed population of NK cells and ILC1, at least in the colon.

### Cytokine Production by Rat ILCs

**Figure 3:**
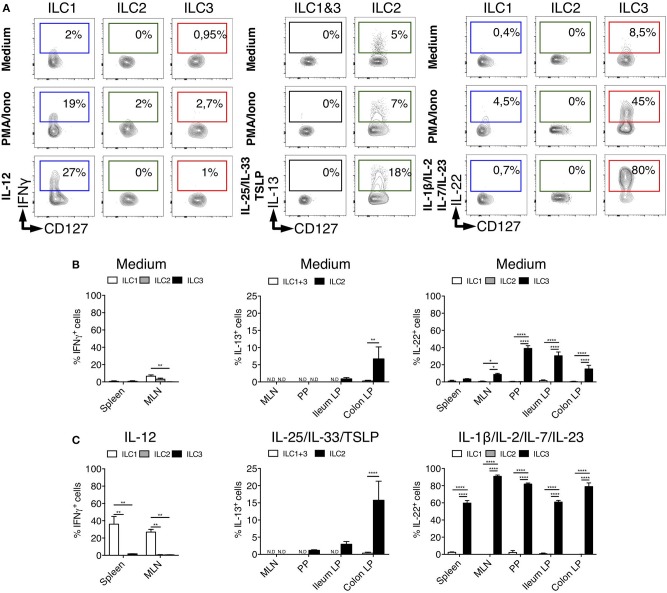
Tissues quantification of cytokine production by ILC subsets in rats. **(A)** Representative intracellular staining for IFNγ (in spleen from LEW rats), IL-22 and IL-13 (in Colon LP from SPD rats) in ILC subsets as defined in Figure 1A after 4 h of culture in the absence (medium) or the presence of PMA/Ionomycin or IL-12, a cocktail of IL-25, IL-33, and TSLP or a cocktail of IL-1β, IL-2, IL-7, and IL-23. Flow cytometry quantification of cytokine-producing cells among ILC subset in the depicted tissues after 4 h of culture in the absence **(B)** or the presence **(C)** of indicated cytokine cocktails. Data are representative of 2 (PMA+ionomycin) to 6 (cytokines) independent experiments, numeric data represents means ± SEM, ND, not detected. ^*^p < 0.05, ^**^p < 0.01, ^****^p < 0.0001.

Human and mouse ILCs are characterized by their specialized production of cytokines, mimicking the profiles observed for CD4^+^ helper T cell subsets (42). To assess whether such a functional specialization also existed for rat ILCs, we analyzed the production of ILC1, ILC2, and ILC3 archetypal cytokines. Because of their low frequency in intestinal tissues at steady state (Figure 1G), we limited our analysis of IFNγ production by ILC1 to the spleen and MLN. Upon activation by PMA and ionomycin, ILC1 (in spleen), ILC2 and ILC3 (in colon LP) selectively produced IFNγ, IL-13, and IL-22, respectively (Figure 3A), while little production of IL-17A was detected especially in colon LP (Supplementary Figures 2A, 3). Contrary to ILCs, however, LP CD4^+^ T cells stimulated with the same conditions strongly produced IL-17A (Supplementary Figure 3). Analysis of unstimulated cells also confirmed the selective production of IL-13 and IL-22 by ILC2 and ILC3, respectively (5, 43, 44) (Figure 3). Reminiscent of *Il5* expression observed in a small subset of colonic ILC2 (Supplementary Figure 2A), the constitutive production of IL-13 was restricted to a small fraction of ILC2 in colon LP and to a lower extent ileum LP, while it was not detected in SLO (Figure 3B). The constitutive production of IL-22 by ILC3 was mostly detected in intestinal tissues (Figure 3B) and restricted to a small fraction of colonic ILC3 at the transcriptional level (Supplementary Figure 2A).

The production of cytokines by ILCs in tissues is mostly triggered by specific cytokines derived from myeloid, epithelial and stromal cells (45). As expected, IL-12 induced IFNγ only in ILC1 while IL-25, IL-33, and TSLP induced IL-13 production only in ILC2 (Figures 3A,C). Quite surprisingly, while cytokine stimulation induced IL-13 production by a fraction of ILC2 in the colon LP, and to a lower extent in the ileum LP, no induction was observed in ILC2 from MLN, suggesting potential distinct patterns of cytokine receptor expression between ILC2 in the LP and in SLO (Figure 3C). Finally, concordant with the selective expression of IL-23R and IL-1R by human and mouse ILC3 verified in our scRNAseq data (Supplementary Figure 2B), a cytokine cocktail containing IL-23 and IL-1β specifically induced a large majority of rat ILC3 from SLO and LP to produce IL-22 (Figures 3A,C).

Importantly, although rat ILC2 expressed low levels of RORγt in LP, MLN and PP, they did not produce IL-22 or IL-17A, even when stimulated with ILC3-stimulating cytokines (Supplementary Figure 3).

### CD4, MHC-II, and NKp46 Identify Distinct Rat ILC3 Subsets

**Figure 4:**
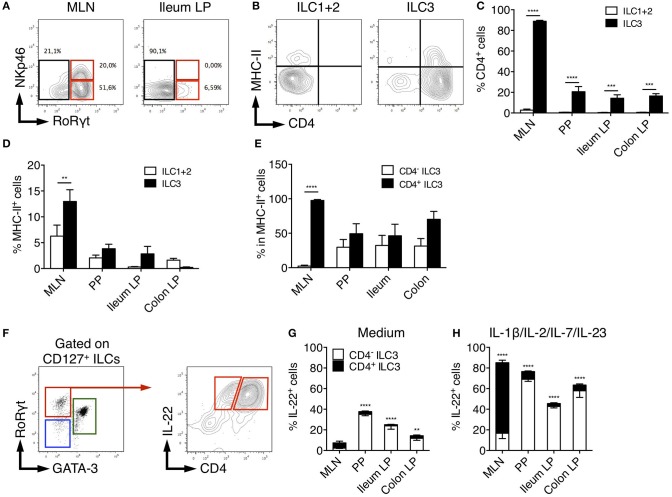
Characterization of rat ILC3 subsets. **(A)** Representative flow cytometry plots showing NKp46 and RORγt expression among total ILC cells isolated from the MLN and ileum LP of SPD rat **(B)** Representative flow cytometry plots showing CD4 and MHC-II expression by ILC1/2 and ILC3 cells isolated from the MLN of SPD rat. **(C)** The percentage of CD4^+^ and **(D)** MHC-II^+^ cells among ILC1/2 (white bars) or ILC3 (black bars) isolated from the indicated tissues of SPD rat. **(E)** The percentage of MHC-II^+^ cells amongst CD4^−^ (white bars) and CD4^+^ (black bars) ILC3 cells. **(F)** Representative flow cytometry plots showing the gating strategy to identify CD4^−^ and CD4^+^ IL-22-producing cells among ILC3 isolated from the colon LP of SPD rat. **(G)** Frequencies of the CD4^+^ and CD4^−^ cells amongst IL-22 producing ILC3 after 4 h of culture in the absence **(G)** or the presence of the indicated stimulating cytokines **(H)**. *n* = 6 per each tissue. ^**^p < 0.01, ^***^p < 0.001, ^****^p < 0.0001.

In adult mice, ILC3 can be separated in developmentally distinct NCR^+^ ILC3 and LTi-like ILC3 based on NKp46 expression (8). We therefore assessed NKp46 expression among rat ILCs in the different tissues. In MLN, a significant fraction (30%) of ILC3 expressed low levels of NKp46 consistent with previous studies (46). In agreement with our scRNAseq data suggesting that all colonic ILC3 were NKp46^−^ CCR6^+^ LTi-like cells (Figure 2C and Supplementary Figure 2B), no NKp46 expression was detected in LP ILC3 (Figure 4A). Mouse LTi-like cells can also express CD4, at least in part (8). Accordingly, we detected CD4 expression in a subset of rat ILC3, but not in ILC1 or ILC2 (Figures 4B,C). Concordant with mouse findings (46), the proportion of CD4^+^ LTi-like ILC3 was the highest in MLN, where they constituted the majority of ILC3 (>80% of total ILC3), whereas CD4^−^ ILC3 dominated the ILC3 composition (>70%) in PP, ileum, and colon LP (Figure 4C). MHC-II^+^ murine RORγt^+^ ILC3 have been recently proposed to contribute to the establishment of gut microbiota tolerance by inducing antigen-specific CD4^+^ T cells apoptosis through the presentation of commensal bacteria-derived peptides (46, 47). Accordingly, a fraction of rat ILC3 expressed MHC-II and we also observed some MHC-II expression in the ILC1/ILC2 fraction (Figures 4B,D), likely due to the expression of MHC-II by ILC2 as previously reported in mice (48). Similar to mice, MHC-II expression was restricted to the CD4^+^ population of LTi-like ILC3 in rat MLN (46). However, this was not the case in PP, ileum and colon LP tissues in which MHC-II was detected in both CD4^−^ and CD4^+^ ILC3 (Figure 4E).

Finally, we compared the production of IL-22 between CD4^+^ and CD4^−^ ILC3 (Figure 4F). Constitutive production of IL-22 was mainly detected in CD4^+^ LTi-like ILC3 in MLN and CD4^−^ ILC3 in PP, ileum and colon LP (Figure 4G). In mice, CD4^+^ ILC3 are considered the most mature LTi-like population and the dominant source of IL-22 in MLN in response to IL-23 (46) and *Citrobacter rodentium* infection (49, 50). Upon stimulation by IL-23 together with IL-1β, IL-2, IL-7, rat CD4^+^ LTi-like cells were indeed the major producers of IL-22 in MLN. However, an opposite pattern was observed in PP, ileum and colon LP (Figure 4H).

### Distribution and Function of ILCs in Different Rat Strains

**Figure 5:**
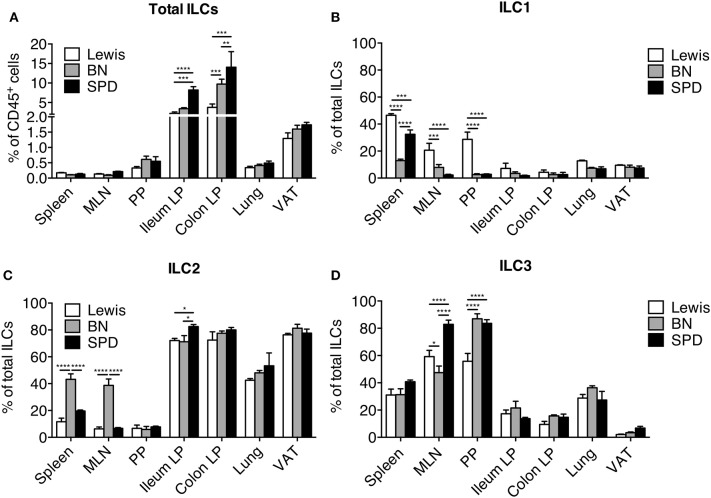
Tissue distribution of ILC populations between rat strains. **(A)** Frequencies of total Lin^−^ CD127^+^ ILCs expressed as percentage of CD45^+^ leukocytes in different tissues from LEW (white bars), BN (gray bars) and SPD (black bars) rats **(B–D)** Frequencies of ILC1 **(B)**, ILC2 **(C)**, and ILC3 **(D)** among total ILCs within each rat strain in different tissues. *n* = 6 per each tissue and rat strain except lung and VAT (*n* = 4). Numeric data represents means ± SEM, significance was determined at *P* < 0.05. ^*^p < 0.05, ^**^p < 0.01, ^***^p < 0.001, ^****^p < 0.0001.

The observation that ILC2 was the dominant ileal and colonic LP ILC population in SPD rats was unexpected (Figures 1A,G). To assess whether this was a peculiarity of the SPD rat strain, we extended our ILC analysis to Lewis (LEW) and Brown Norway (BN) (Supplementary Figure 4) rats, which are known to exhibit genetically-driven different Th cell response profiles (51). Higher frequencies of total ILCs in ileum and colon LP compared to SLO were verified for all strains (Figure 5A). Frequencies of total gut ILCs were slightly higher in SPD as compared to LEW and BN rats. In addition, strain-specific differences between ILC subset frequencies were also observed: ILC1 were enriched in SLO from LEW rats as compared to SPD and BN (Figure 5B); ILC2 were enriched in spleen and MLN of BN as compared to SPD and LEW rats (Figure 5C); SPD rats exhibited higher frequencies of ILC3 in MLN as compared to the two other strains (Figure 5D). Furthermore, in tissues known to harbor a majority of ILC2 such as lungs and adipose tissue (37, 52), ILC subtype frequencies were similar between the different rat strains (Figure 5). In all three rat strains analyzed, ILC3 were thus predominant in SLOs while ILC2 dominated the ILC pool in lungs, visceral adipose tissue (VAT), ileal and colonic LP.

**Figure 6:**
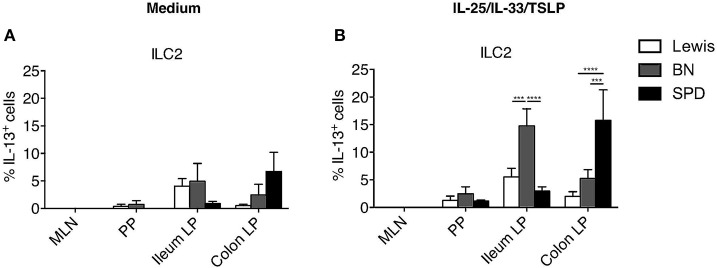
Comparison of ILC2-derived IL-13 production between rat strains. **(A)** The percentage of constitutive ILC2-derived IL-13 production in the indicated tissues of LEW (white bars), BN (gray bars) and SPD (black bars) rats and **(B)** after 4 h of stimulation with ILC2 specific cocktail. (*n* = 6) per each tissue and rat strain. Numeric data represents means ± SEM, significance was determined at *P* < 0.05. ^***^p < 0.001, ^****^p < 0.0001.

We next compared IL-13 production by ILC2 in the three rat strains. With our stimulation cocktail (IL-25, IL-33, and TSLP), IL-13 production by ILC2 was extremely low in PP, and not detectable in MLN whatever the rat strain (Figures 6A,B). In the intestine, ileum ILC2 produced more IL-13 in BN rats than in LEW and SPD, whereas colonic ILC2 produced more IL-13 in SPD (Figures 3A,B, 6B). Taken together, these data suggest that even if ILC2 are largely and equally predominant in ileum and colon LP of all rat strains analyzed, their function exhibit strain and intestinal segment dependent differences.

### Sex-Dependent Frequencies of ILC2

**Figure 7:**
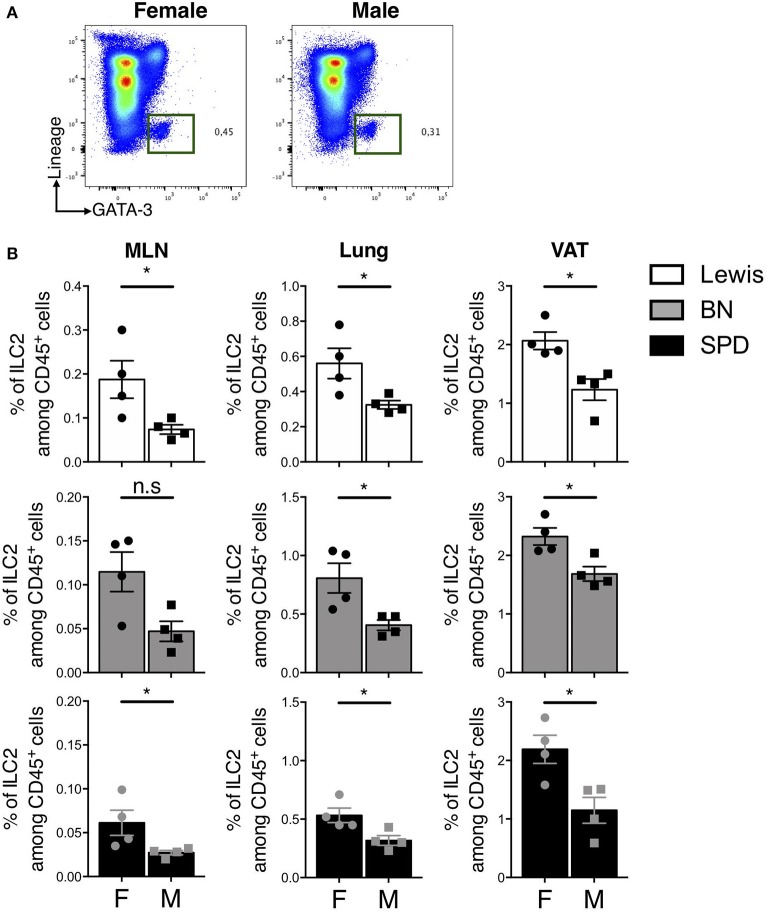
Sex-effect on ILC2 proportions in MLN, lung and VAT of rats. **(A)** Representative plots of Lin^−^ GATA-3^+^ (Pregated on live CD45^+^ cells) ILC2 in the lung of SPD female vs. male rats. **(B)** ILC2 frequencies in MLN, lung and visceral adipose tissues (VAT) of different rat strains. (*n* = 4), numeric data represents means ± SEM, statistical differences were determined by nonparametric Mann Whitney test with significance was determined. F, Female; M, Male. ^*^p < 0.05, n.s, not significant.

Finally, we sought to determine whether sex-dependent differences of ILC2 frequencies previously reported in mice (53) also held true in rats. In line with previous results, the expression of androgen receptor (AR) was restricted to ILC2 in our scRNAseq data (Supplementary Figure 2B). Androgen signaling was shown to limit ILC2 proliferation in mouse males (53). We did not observe different frequencies of total ILCs and ILC2 between male and female rats in the gut (data not shown). However, higher frequencies of ILC2 were detected in MLN, lungs and VAT of females as compared to males independently of the strain (Figures 7A,B).

## Discussion

In this study, we characterized ILC populations in rat intestinal tissues and SLO. We first confirmed that ILCs were strongly enriched in rat ileal and colonic LP. Our study showed that ILC3 could be separated into NCR^+^ ILC3 and LTi-like ILC3, a fraction of which expressed CD4. Both subtypes were present in SLOs, where regardless of the strain, ILC3 were the dominant ILC population, while only LTi-like ILC3 were detected in the intestinal LP. The main producers of IL-22 were LTi-like ILC3 in MLN and CD4^−^ ILC3 in the intestine. As in mice and humans, rat ILC2 produced IL-13 constitutively and in response to stimulation by IL-25, IL-33, and TSLP. Interestingly and contrasting with previous data in mice and human (54), we identified ILC2 as the dominant ILC subset in rat ileal and colonic LP (37), a finding we confirmed and extended using unbiased scRNAseq profiling.

ILC2 are considered to be a homogeneous subset, yet differences in surface marker expression and tissue distribution have been recently reported (55). Several hypotheses can explain the distribution differences of intestinal ILCs between rats and mice. Since high frequencies of ILC2 were observed in the gut LP but not MLN and PP, this could reflect distinct responses to intestinal microbiota and/or food composition. In our study, ILC2 dominance in the rat intestine was observed regardless of animal's origin (commercial vs. bred and maintained in our animal facility), thus excluding any bias due to variable microbiota or infection generated in our facility. A recent study from Locksley's group actually showed that numbers of tissue ILC2 were preserved in germ-free mice, suggesting that endogenous tissue- rather than microbiota-derived signals drive the maturation of ILC2, thus anticipating tissue-specific perturbations occurring later in life (56). However, it was shown that infection with the intestinal worm *Nippostongylus brasiliensis* triggers epithelial cells to release IL-25 and IL-33, which in turn cause ILC2 expansion in mice (57). Repeated injection of IL-25 in mice was shown to promote the expansion of so-called KLRG1^high^ inflammatory ILC2 (iILC2) in lung, MLN, spleen and liver (58). However, these iILC2 were virtually absent in untreated mice. Whether the strong enrichment of ILC2 in intestinal tissue in rats is dependent on IL-25 and IL-33 levels remains to be determined.

An interesting feature of rat ILC2 is their intermediate expression level of RORγt, mirroring those seen in mouse MLN and lungs after *N. brasiliensis* infection (48). Similar iILC2 could also be induced by injection of IL-25 and their low levels of RORγt expression was associated with their capacity to produce IL-17A in Th17 conditions (48). Such mixed ILC2/ILC3-like subsets producing IL-5, IL-17A as well IL-22 were also described *in vivo* in a model of skin inflammation induced by IL-23 in mice (59). In addition, ILC2 with ILC3-like features were recently described in human and appeared to be present in the blood of healthy volunteers (60). However, rat ILC2 from the colon LP and SLO did not produce IL-22 or IL-17 even when stimulated with a cytokine cocktail known to promote ILC3 activation, suggesting that LP ILC2 do not exhibit ILC3-like features. This absence of ILC3-like features in ILC2 was further confirmed by our scRNAseq profiling of total colon ILC. Whether prolonged stimulation of rat LP ILC2 with Th17 inducing cytokines could promote ILC3-related cytokine (60) remains to be addressed.

Regarding the opposite effect of micronutrient environment, a previous report indicated that a deficiency in retinoic acid (RA) severely diminished the frequency of ILC3 and paradoxically resulted in dramatic expansion of ILC2s in mice (14). However, such a deficiency is unlikely to explain ILC2 increased levels in rats as 1. rats were fed regular diet; 2. ILC3 were largely predominant in MLN and PP as previously shown in mice; 3. both ILC2 and ILC3 appeared to proliferate similarly in the ileal LP (Supplementary Figure 5).

We then speculated that differences between rats and mice or humans would be related to genetics. Different genetic backgrounds between rodent strains are known to affect immune cells frequencies, immune responses and further tissue localization (51). By assessing the tissue composition of ILC subsets in three rat strains, we revealed a specific distribution reflecting what was previously described for T cells. In fact, LEW and BN rats are known to harbor distinct immune response polarization in SLO. LEW rats are susceptible to Th1-mediated autoimmune diseases, while BN rats are highly susceptible to Th2-mediated autoimmune disease (51). Interestingly, we found that ILC1 were the major population of LEW rats ILCs, specifically in SLO. Likewise, spleen and MLN from BN rats contained high frequency of ILC2. These data suggest that the Th1 vs. Th2 prone feature of LEW vs. BN rat respectively, which is known to be genetically determined (61), might also relate to the relative abundance of ILC1 and ILC2 in these strains.

We also observed that spleen and MLN of SPD rats contained higher frequencies of ILC3 among total ILCs compared to LEW or BN rats. It is worth pointing out that this observation was consolidated by the fact that ILC3 subset in this strain produced higher IL-22 in the SLO compared to LEW and BN strains. Whether these differences are strain specific or related to the fact that SPD rats are outbred as compared to LEW and BN, remains to be determined. However, ILCs distribution could be more likely governed by rat specific microbial or environmental factors in the intestine as a large pre-dominance of the ILC2 population was observed in all strains.

By providing the first description of rat ILCs, including a deep transcriptomic characterization in the colon by scRNAseq, our study provides a useful resource for the community. Although ILCs appear remarkably conserved between species regarding their phenotype, function and differentiation, we suggest that relative abundance of ILC subsets at mucosal sites might differ between species likely in response to both genetic and environmental factors. Whether the enrichment of ILC2 in the gut LP of rats has an impact on immune responses to helminths, bacteria (62) or intestinal inflammation (63) deserves further investigations.

## Data Availability Statement

Raw sequencing reads of scRNA-seq data as well as UMI tables are available on the Gene Expression Omnibus under accession number GEO: GSE143920.

## Ethics Statement

The animal study was reviewed and approved by EU Directive 2010/63/EU for animal experiments.

## Author Contributions

AA, JP, RJ, and JM designed the study. AA performed most of the experiments and graphed the data. TL performed scRNA-seq analysis. GB, LB-D, CF, and CL participated in experimental procedures. AA, GB, RT-M, JP, RJ, and JM analyzed the data and wrote the manuscript.

### Conflict of Interest

The authors declare that the research was conducted in the absence of any commercial or financial relationships that could be construed as a potential conflict of interest.

## Abbreviations

ILCs: Innate lymphoid cells
IL-22: Interleukin-22
TSLP: Thymic stromal lymphopoietin
MLN: mesenteric lymph nodes
PP: Peyer's patches
LP: Lamina propria
SPD: Sprague Dawley
BN: Brown Norway.

## References

[1] Hazenberg MD Spits H. Human innate lymphoid cells. Blood. (2014) 124:700–9. 10.1182/blood-2013-11-427781 24778151

[2] Spits H Cupedo T. Innate lymphoid cells: emerging insights in development, lineage relationships, and function. Annu Rev Immunol. (2012) 30:647–75. 10.1146/annurev-immunol-020711-075053 22224763

[3] Bernink JH Peters CP Munneke M te Velde AA Meijer SL Weijer K. Human type 1 innate lymphoid cells accumulate in inflamed mucosal tissues. Nat Immunol. (2013) 14:221–9. 10.1038/ni.2534 23334791

[4] Huang Y Paul WE. Inflammatory group 2 innate lymphoid cells. Int Immunol. (2016) 28:23–8. 10.1093/intimm/dxv044 26232596 PMC4715228

[5] Chen L He Z Slinger E Bongers G Lapenda TLS Pacer ME. IL-23 activates innate lymphoid cells to promote neonatal intestinal pathology. Mucosal Immunol. (2015) 8:390–402. 10.1038/mi.2014.77 25160819 PMC4326561

[6] Montaldo E Juelke K Romagnani C. Group 3 innate lymphoid cells (ILC3s): origin, differentiation, and plasticity in humans and mice. Eur J Immunol. (2015) 45:2171–82. 10.1002/eji.201545598 26031799

[7] Kim CH Hashimoto-Hill S Kim M. Migration and tissue tropism of innate lymphoid cells. Trends Immunol. (2016) 37:68–79. 10.1016/j.it.2015.11.003 26708278 PMC4744800

[8] Melo-Gonzalez F Hepworth MR. Functional and phenotypic heterogeneity of group 3 innate lymphoid cells. Immunology. (2017) 150:265–75. 10.1111/imm.12697 27935637 PMC5290240

[9] Wang S Xia P Chen Y Qu Y Xiong Z Ye B. Regulatory innate lymphoid cells control innate intestinal inflammation. Cell. (2017) 171:201–16.e18. 10.1016/j.cell.2017.07.027 28844693

[10] Artis D Spits H. The biology of innate lymphoid cells. Nature. (2015) 517:293–301. 10.1038/nature14189 25592534

[11] Tomasello E Bedoui S. Intestinal innate immune cells in gut homeostasis and immunosurveillance. Immunol Cell Biol. (2013) 91:201–3. 10.1038/icb.2012.85 23478396

[12] Goverse G Labao-Almeida C Ferreira M Molenaar R Wahlen S Konijn T. Vitamin A controls the presence of RORγ+ innate lymphoid cells and lymphoid tissue in the small intestine. J Immunol. (2016) 196:5148–55. 10.4049/jimmunol.1501106 27183576

[13] Sawa S Cherrier M Lochner M Satoh-Takayama N Fehling HJ Langa F. Lineage relationship analysis of RORgammat+ innate lymphoid cells. Science. (2010) 330:665–9. 10.1126/science.1194597 20929731

[14] Spencer SP Wilhelm C Yang Q Hall JA Bouladoux N Boyd A. Adaptation of innate lymphoid cells to a micronutrient deficiency promotes type 2 barrier immunity. Science. (2014) 343:432–7. 10.1126/science.1247606 24458645 PMC4313730

[15] Nagpal R Wang S Solberg Woods LC Seshie O Chung ST Shively CA. Comparative microbiome signatures and short-chain fatty acids in mouse, rat, non-human primate, and human feces. Front Microbiol. (2018) 9:2897. 10.3389/fmicb.2018.02897 30555441 PMC6283898

[16] Chung H Pamp SJ Hill JA Surana NK Edelman SM Troy EB. Gut immune maturation depends on colonization with a host-specific microbiota. Cell. (2012) 149:1578–93. 10.1016/j.cell.2012.04.037 22726443 PMC3442780

[17] Jacob HJ. Functional genomics and rat models. Genome Res. (1999) 9:1013–6. 10.1101/gr.9.11.1013 10568741

[18] Aitman TJ Critser JK Cuppen E Dominiczak A Fernandez-Suarez XM Flint J. Progress and prospects in rat genetics: a community view. Nat Genet. (2008) 40:516–22. 10.1038/ng.147 18443588

[19] Hajj Hussein I-A Tohme R Barada K Mostafa MH Freund J-N Jurjus RA. Inflammatory bowel disease in rats: bacterial and chemical interaction. World J Gastroenterol. (2008) 14:4028–39. 10.3748/wjg.14.4028 18609687 PMC2725342

[20] Aleixandre de Artiñano A Miguel Castro M. Experimental rat models to study the metabolic syndrome. Br J Nutr. (2009) 102:1246–53. 10.1017/S0007114509990729 19631025

[21] Wong SK Chin K-Y Suhaimi FH Fairus A Ima-Nirwana S. Animal models of metabolic syndrome: a review. Nutr Metab. (2016) 13:65. 10.1186/s12986-016-0123-9 27708685 PMC5050917

[22] Stuart T Butler A Hoffman P Hafemeister C Papalexi E Mauck WM. Comprehensive Integration of Single-Cell *Data*. Cell. (2019) 177:1888–902.e21. 10.1016/j.cell.2019.05.031 31178118 PMC6687398

[23] Becht E McInnes L Healy J Dutertre C-A Kwok IWH Ng LG Dimensionality reduction for visualizing single-cell data using UMAP. Nat Biotechnol. (2018) 37:38–44. 10.1038/nbt.4314 30531897

[24] Martin JC Chang C Boschetti G Ungaro R Giri M Grout JA. Single-cell analysis of Crohn's disease lesions identifies a pathogenic cellular module associated with resistance to anti-TNF therapy. Cell. (2019) 178:1493–508.e20. 10.1016/j.cell.2019.08.008 31474370 PMC7060942

[25] Björklund ÅK Forkel M Picelli S Konya V Theorell J Friberg D. The heterogeneity of human CD127(+) innate lymphoid cells revealed by single-cell RNA sequencing. Nat Immunol. (2016) 17:451–60. 10.1038/ni.3368 26878113

[26] Cortez VS Colonna M. Diversity and function of group 1 innate lymphoid cells. Immunol Lett. (2016) 179:19–24. 10.1016/j.imlet.2016.07.005 27394699 PMC5658203

[27] Borroto A Lama J Niedergang F Dautry-Varsat A Alarcón B Alcover A. The CD3 epsilon subunit of the TCR contains endocytosis signals. J Immunol. (1999) 163:25–31. 10384095

[28] Martin-Blanco N Jiménez Teja D Bretones G Borroto A Caraballo M Screpanti I. CD3ε recruits numb to promote TCR degradation. Int Immunol. (2016) 28:127–37. 10.1093/intimm/dxv060 26507128

[29] Vosshenrich CAJ García-Ojeda ME Samson-Villéger SI Pasqualetto V Enault L Richard-Le Goff O. A thymic pathway of mouse natural killer cell development characterized by expression of GATA-3 and CD127. Nat Immunol. (2006) 7:1217–24. 10.1038/ni1395 17013389

[30] Allan DSJ Cerdeira AS Ranjan A Kirkham CL Aguilar OA Tanaka M. Transcriptome analysis reveals similarities between human blood CD3- CD56bright cells and mouse CD127+ innate lymphoid cells. Sci Rep. (2017) 7:3501. 10.1038/s41598-017-03256-0 28615725 PMC5471261

[31] Colonna M. Innate lymphoid cells: diversity, plasticity, and unique functions in immunity. Immunity. (2018) 48:1104–17. 10.1016/j.immuni.2018.05.013 29924976 PMC6344351

[32] Mjösberg JM Trifari S Crellin NK Peters CP van Drunen CM Piet B. Human IL-25- and IL-33-responsive type 2 innate lymphoid cells are defined by expression of CRTH2 and CD161. Nat Immunol. (2011) 12:1055–62. 10.1038/ni.2104 21909091

[33] Walker JA Barlow JL McKenzie ANJ. Innate lymphoid cells–how did we miss them? Nat Rev Immunol. (2013) 13:75–87. 10.1038/nri3349 23292121

[34] Longman RS Diehl GE Victorio DA Huh JR Galan C Miraldi ER. CX3CR1+ mononuclear phagocytes support colitis-associated innate lymphoid cell production of IL-22. J Exp Med. (2014) 211:1571–83. 10.1084/jem.20140678 25024136 PMC4113938

[35] Guo X Qiu J Tu T Yang X Deng L Anders RA. Induction of innate lymphoid cell-derived interleukin-22 by the transcription factor STAT3 mediates protection against intestinal infection. Immunity. (2014) 40:25–39. 10.1016/j.immuni.2013.10.021 24412612 PMC3919552

[36] Simoni Y Fehlings M Kløverpris HN McGovern N Koo S-L Loh CY. Human innate lymphoid cell subsets possess tissue-type based heterogeneity in phenotype and frequency. Immunity. (2017) 46:148–61. 10.1016/j.immuni.2016.11.005 27986455 PMC7612935

[37] Dutton EE Camelo A Sleeman M Herbst R Carlesso G Belz GT. Characterisation of innate lymphoid cell populations at different sites in mice with defective T cell immunity. Wellcome Open Res. (2017) 2:117. 10.12688/wellcomeopenres.13199.1 29588921 PMC5854988

[38] Robinette ML Fuchs A Cortez VS Lee JS Wang Y Durum SK. Transcriptional programs define molecular characteristics of innate lymphoid cell classes and subsets. Nat Immunol. (2015) 16:306–17. 10.1038/ni.3094 25621825 PMC4372143

[39] Califano D Cho JJ Uddin MN Lorentsen KJ Yang Q Bhandoola A. Transcription factor Bcl11b controls identity and function of mature type 2 innate lymphoid cells. Immunity. (2015) 43:354–68. 10.1016/j.immuni.2015.07.005 26231117 PMC4657441

[40] Withers DR Hepworth MR Group 3 innate lymphoid cells: communications hubs of the intestinal immune system. Front Immunol. (2017) 8:1298 10.3389/fimmu.2017.01298 29085366 PMC5649144

[41] Penny HA Hodge SH Hepworth MR. Orchestration of intestinal homeostasis and tolerance by group 3 innate lymphoid cells. Semin Immunopathol. (2018) 40:357–70. 10.1007/s00281-018-0687-8 29737384 PMC6060788

[42] Vivier E Artis D Colonna M Diefenbach A Di Santo JP Eberl G. Innate lymphoid cells: 10 years on. Cell. (2018) 174:1054–66. 10.1016/j.cell.2018.07.017 30142344

[43] Cella M Fuchs A Vermi W Facchetti F Otero K Lennerz JKM. A human natural killer cell subset provides an innate source of IL-22 for mucosal immunity. Nature. (2009) 457:722–5. 10.1038/nature07537 18978771 PMC3772687

[44] Fallon PG Ballantyne SJ Mangan NE Barlow JL Dasvarma A Hewett DR. Identification of an interleukin (IL)-25-dependent cell population that provides IL-4, IL-5, and IL-13 at the onset of helminth expulsion. J Exp Med. (2006) 203:1105–16. 10.1084/jem.20051615 16606668 PMC2118283

[45] Mortha A Burrows K. Cytokine networks between innate lymphoid cells and myeloid cells. Front Immunol. (2018) 9:191. 10.3389/fimmu.2018.00191 29467768 PMC5808287

[46] Mackley EC Houston S Marriott CL Halford EE Lucas B Cerovic V. CCR7-dependent trafficking of RORγ+ ILCs creates a unique microenvironment within mucosal draining lymph nodes. Nat Commun. (2015) 6:5862. 10.1038/ncomms6862 25575242 PMC4354100

[47] Hepworth MR Fung TC Masur SH Kelsen JR McConnell FM Dubrot J. Immune tolerance. Group 3 innate lymphoid cells mediate intestinal selection of commensal bacteria-specific CD4+ T cells. Science. (2015) 348:1031–5. 10.1126/science.aaa4812 25908663 PMC4449822

[48] Oliphant CJ Hwang YY Walker JA Salimi M Wong SH Brewer JM. MHCII-mediated dialog between group 2 innate lymphoid cells and CD4(+) T cells potentiates type 2 immunity and promotes parasitic helminth expulsion. Immunity. (2014) 41:283–95. 10.1016/j.immuni.2014.06.016 25088770 PMC4148706

[49] Zhong C Zheng M Zhu J. Lymphoid tissue inducer-A divergent member of the ILC family. Cytokine Growth Factor Rev. (2018) 42:5–12. 10.1016/j.cytogfr.2018.02.004 29454785 PMC6089680

[50] Sonnenberg GF Monticelli LA Elloso MM Fouser LA Artis D. CD4(+) lymphoid tissue-inducer cells promote innate immunity in the gut. Immunity. (2011) 34:122–34. 10.1016/j.immuni.2010.12.009 21194981 PMC3035987

[51] Fournié GJ Cautain B Xystrakis E Damoiseaux J Mas M Lagrange D. Cellular and genetic factors involved in the difference between Brown Norway and Lewis rats to develop respectively type-2 and type-1 immune-mediated diseases. Immunol Rev. (2001) 184:145–60. 10.1034/j.1600-065x.2001.1840114.x 12086309

[52] Bénézech C Jackson-Jones LH. ILC2 orchestration of local immune function in adipose tissue. Front Immunol. (2019) 10:171. 10.3389/fimmu.2019.00171 30792718 PMC6374325

[53] Laffont S Blanquart E Savignac M Cénac C Laverny G Metzger D. Androgen signaling negatively controls group 2 innate lymphoid cells. J Exp Med. (2017) 214:1581–92. 10.1084/jem.20161807 28484078 PMC5461006

[54] Krämer B Goeser F Lutz P Glässner A Boesecke C Schwarze-Zander C. Compartment-specific distribution of human intestinal innate lymphoid cells is altered in HIV patients under effective therapy. PLoS Pathog. (2017) 13:e1006373. 10.1371/journal.ppat.1006373 28505204 PMC5444854

[55] Neill DR Wong SH Bellosi A Flynn RJ Daly M Langford TKA. Nuocytes represent a new innate effector leukocyte that mediates type-2 immunity. Nature. (2010) 464:1367–70. 10.1038/nature08900 20200518 PMC2862165

[56] Ricardo-Gonzalez RR Van Dyken SJ Schneider C Lee J Nussbaum JC Liang H-E. Tissue signals imprint ILC2 identity with anticipatory function. Nat Immunol. (2018) 19:1093–99. 10.1038/s41590-018-0201-4 30201992 PMC6202223

[57] Licona-Limón P Kim LK Palm NW Flavell RA. TH2, allergy and group 2 innate lymphoid cells. Nat Immunol. (2013) 14:536–42. 10.1038/ni.2617 23685824

[58] Huang Y Guo L Qiu J Chen X Hu-Li J Siebenlist U. IL-25-responsive, lineage-negative KLRG1(hi) cells are multipotential “inflammatory” type 2 innate lymphoid cells. Nat Immunol. (2015) 16:161–9. 10.1038/ni.3078 25531830 PMC4297567

[59] Bielecki P Riesenfeld SJ Kowalczyk MS Vesely MCA Kroehling L Yaghoubi P Skin inflammation driven by differentiation of quiescent tissue-resident ILCs into a spectrum of pathogenic effectors. bioRxiv. (2018) 461228 10.1101/461228

[60] Bernink JH Ohne Y Teunissen MBM Wang J Wu J Krabbendam L. c-Kit-positive ILC2s exhibit an ILC3-like signature that may contribute to IL-17-mediated pathologies. Nat Immunol. (2019) 20:992–1003. 10.1038/s41590-019-0423-0 31263279

[61] Mathieson PW Thiru S Oliveira DB. Regulatory role of OX22high T cells in mercury-induced autoimmunity in the brown Norway rat. J Exp Med. (1993) 177:1309–16. 10.1084/jem.177.5.1309 8478610 PMC2191016

[62] Frisbee AL Saleh MM Young MK Leslie JL Simpson ME Abhyankar MM. IL-33 drives group 2 innate lymphoid cell-mediated protection during Clostridium difficile infection. Nat Commun. (2019) 10:2712. 10.1038/s41467-019-10733-9 31221971 PMC6586630

[63] Camelo A Barlow JL Drynan LF Neill DR Ballantyne SJ Wong SH. Blocking IL-25 signalling protects against gut inflammation in a type-2 model of colitis by suppressing nuocyte and NKT derived IL-13. J Gastroenterol. (2012) 47:1198–211. 10.1007/s00535-012-0591-2 22539101 PMC3501170

