# Supplementary material for: Characterization of Rat ILCs Reveals ILC2 as the Dominant Intestinal Subset
Source: Front Immunol. 2020 Feb 19;11:255. doi: 10.3389/fimmu.2020.00255 (PMC7043102; doi:10.3389/fimmu.2020.00255)
Supplement: Supplementary file 1 [file Data_Sheet_1.docx]

Supplementary Material

## Supplementary Figures


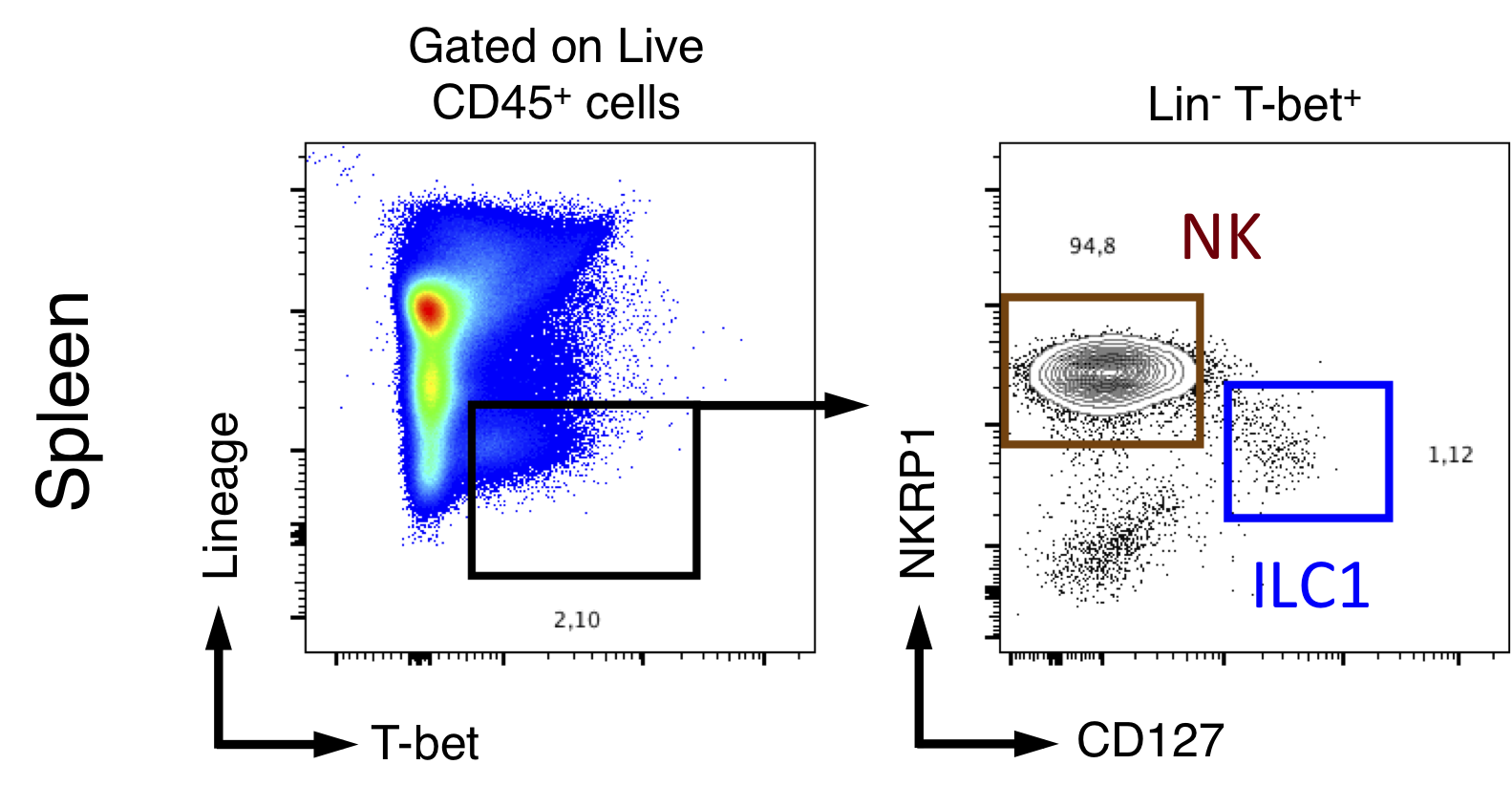


**Supplementary Figure 1.** **Flow cytometry identification of NK cells and ILC1 in rat.**

## Representative flow cytometry plots showing the gating strategy to identify NK cells (Lin^-^ T-bet^+^ NKRP1^+^ CD127^-^ cells) and ILC1 (Lin^-^ T-bet^+^ NKRP1^int^ CD127^+^) in the spleen of LEW rat.

**
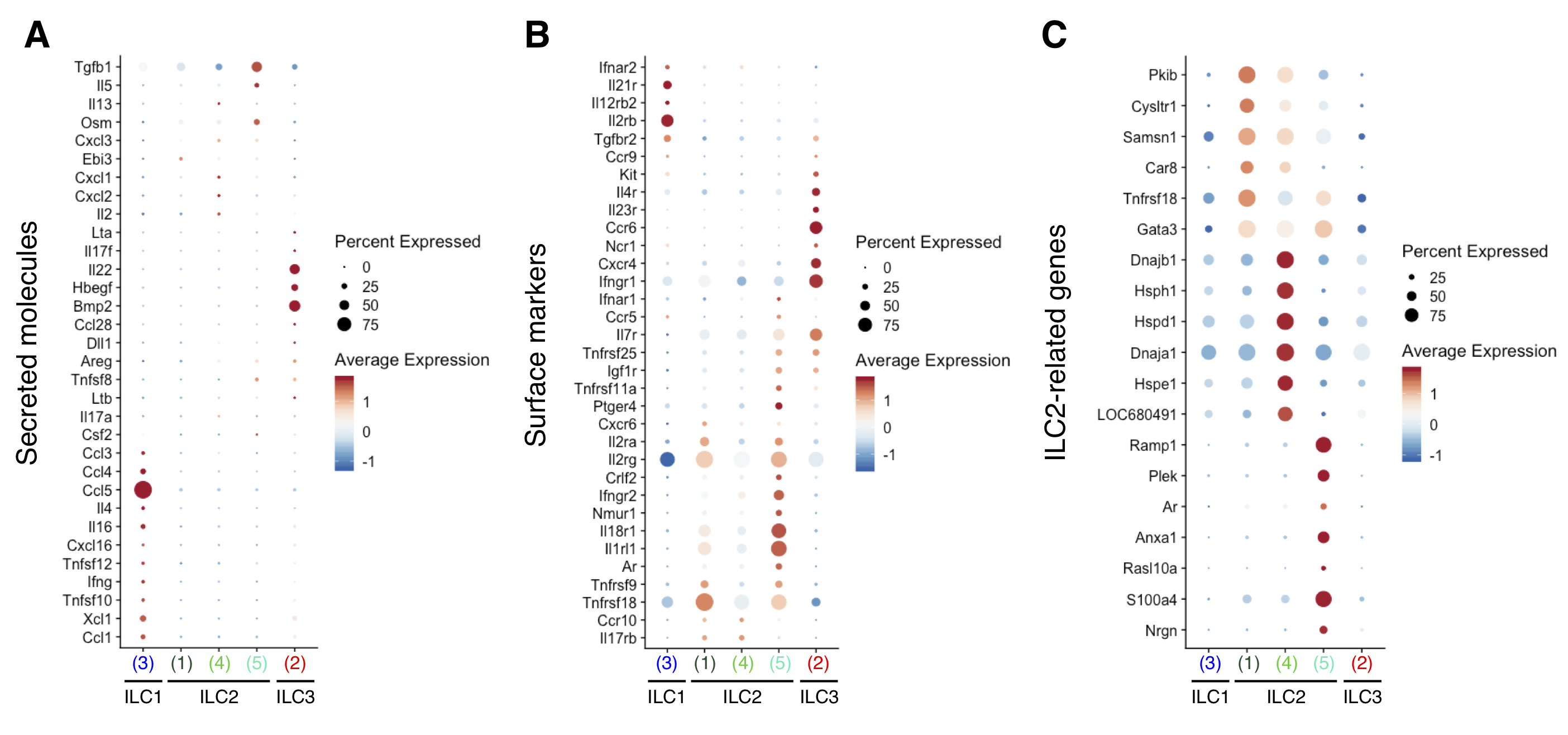
**

**Supplementary Figure 2.** **Expression of selected genes among ILC clusters in the colon LP of rat**.

Dot plot graphs showing 1: the percentage of cells expressing the respective selected marker gene using dot size and 2: the average expression level of that gene based on unique molecular identifier (UMI) counts from scRNAseq analysis on Lin^-^ CD127^+^ cells from Colon LP of 1 SPD rat. Plots shows expression of genes encoding (**A**) secreted molecules, (**B**) surface markers and (**C**) top 6 genes DEs between ILC2 cluster (C1, C4 and C5).


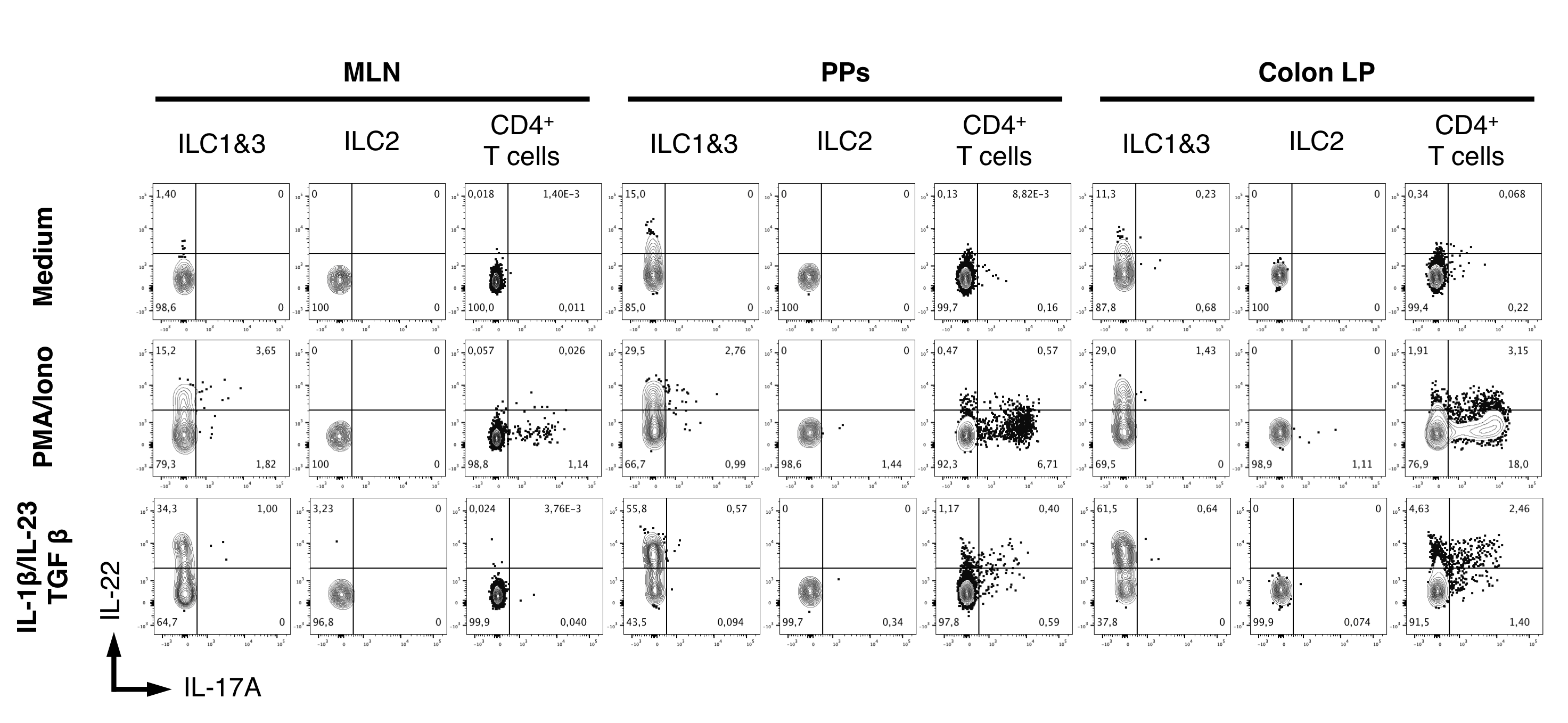


**Supplementary Figure 3.** **Stimulation conditions for cytokine production by rat ILCs**.

Representative IL-17A and IL-22 intracellular staining among GATA-3^-^ ILCs (ILC1+3), GATA3^+^ ILC (ILC2) and GATA3^-^ CD4^+^ T cells from MLN, PP and colon LP of SPD rats cultured for 4 hours either unstimulated (medium) or stimulated by PMA/Ionomycin or a Th17-specific stimulating cytokine cocktail. Shown plots are representative of two independent experiments.

**
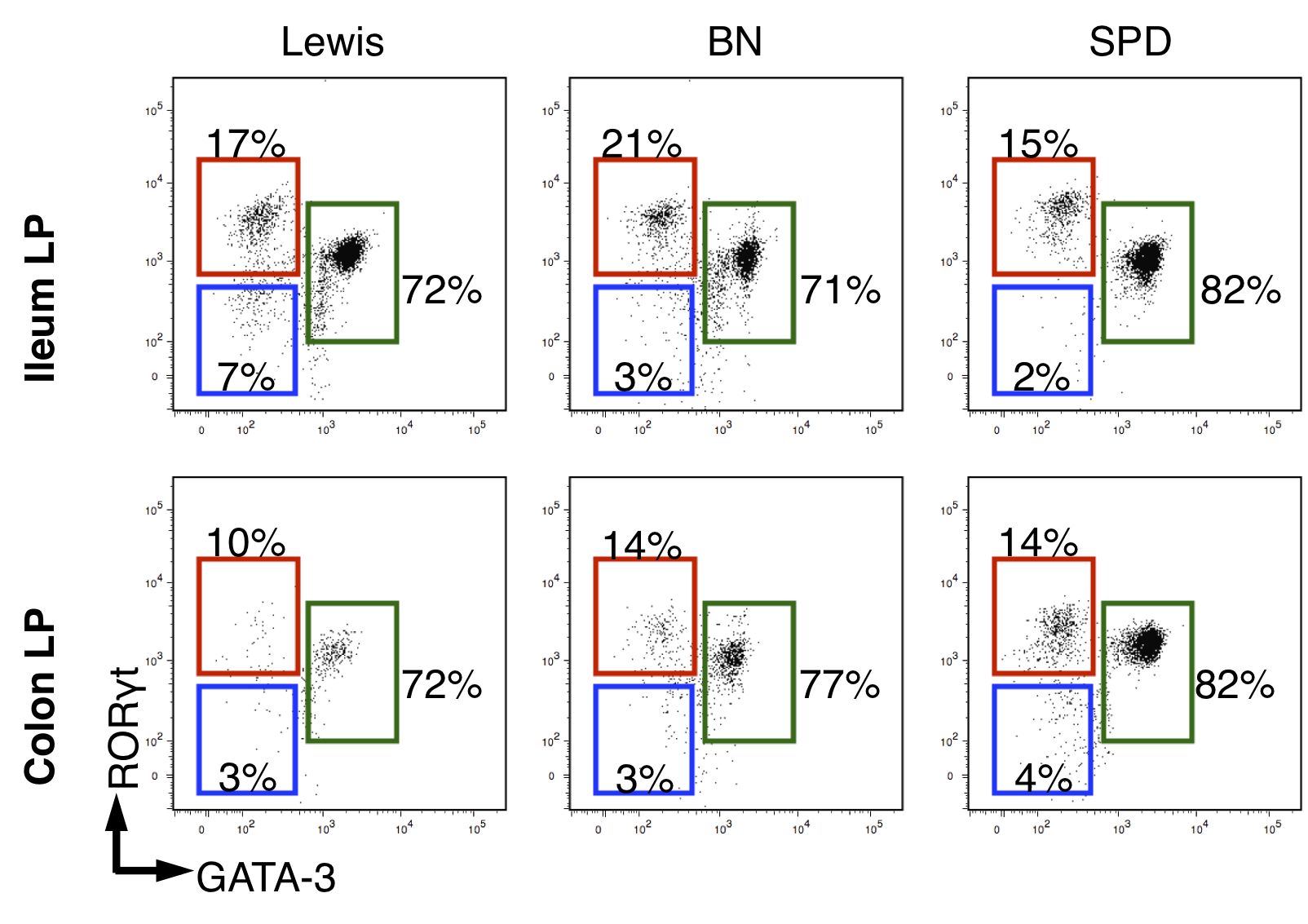
**

**Supplementary Figure 4. Intestinal ILC subsets characterization in different rat strains.**

Representative flow cytometry plots showing the expression of RORγt and GATA-3 in ILC from ileum and colon LP in LEW, BN and SPD rats. Shown plots are representative of six independent experiments.


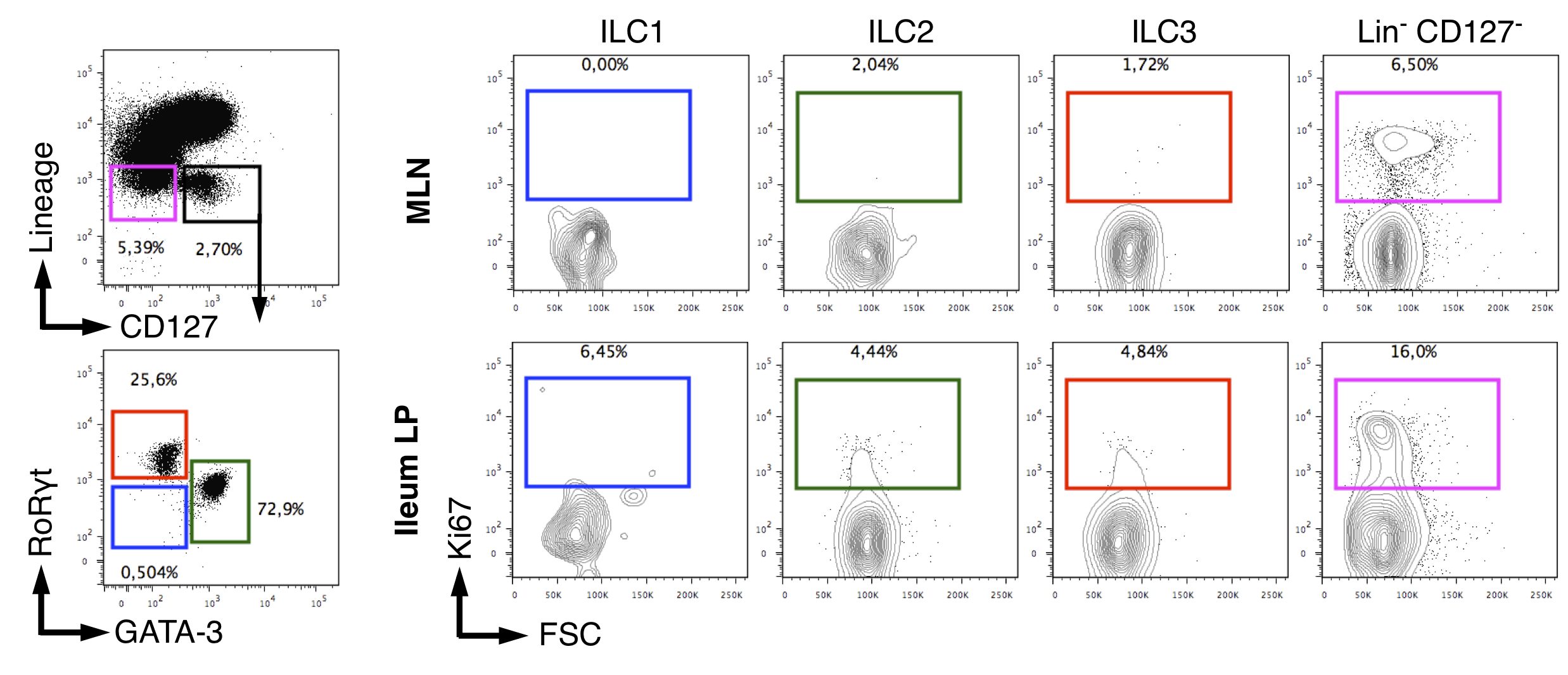


**Supplementary Figure 5. In vivo proliferation of ILC subsets in MLN and Ileum LP of SPD rat.** One representative flow cytometry experiment out of three showing intracellular staining of Ki67 proliferation marker among different ILC subsets and Lin^-^ CD127^-^ cells in MLN and ileum LP of SPD rat.
